# Mid‐term performance of a unicompartmental knee prosthesis

**DOI:** 10.1002/jeo2.70670

**Published:** 2026-02-26

**Authors:** Akbar Nawab, Patricia Bartelt, Ryan Molli, J. Mandume Kerina, Samuel Wellman, Dean Sukin, Stefano Biguzzi, Colleen Nawab, Daniel Hass, Ashton Dukes, Anthony Robins

**Affiliations:** ^1^ Ellis & Badenhausen Orthopedics Louisville Kentucky USA; ^2^ Medacta USA, Clinical Research Franklin Tennessee USA; ^3^ Whole Health Orthopedic Institute Meadville Pennsylvania USA; ^4^ UNOVA Ambulatory Surgery Center Lady Lake Florida USA; ^5^ Orthopaedic Surgery Duke University Medical Center Durham North Carolina USA; ^6^ Alpine Orthopedics and Sports Medicine Bozeman Montana USA; ^7^ Medacta International Clinical Research Castel San Pietro Switzerland; ^8^ Ellis & Badenhausen Clinical Research Louisville Kentucky USA; ^9^ Orthopaedic Surgery & Sports Medicine – Clinical Instructor University of Washington Seattle Washington USA

**Keywords:** medial compartment osteoarthritis, MOTO medial prosthesis, patient‐reported outcomes, survivorship analysis, unicompartmental knee arthroplasty

## Abstract

**Purpose:**

The objective of this study was to report midterm patient‐reported outcomes (PROs) and survivorship of the Medacta MOTO Medial® Unicompartmental prosthesis (*Medacta International*, *Castel San Petro, Switzerland*) and surgical technique.

**Methods:**

This prospective, multicentre study included 207 adults aged 43–85. Of these, 170 reached 2‐year and 90 reached 5‐year follow‐up. PROs included the forgotten joint score (FJS), Oxford knee score (OKS) and knee injury and osteoarthritis outcome score (KOOS) subscales: Pain, Symptoms, ADL, Sport and QOL. Stress radiographs were divided into three types, quantifying the amount of medial space opening and deformity correction. Data were collected at preoperative, intraoperative, 4 weeks, 6 months, 1‐year, 2‐year and 5‐year. Complications, revisions and Kaplan–Meier survivorship were analysed.

**Results:**

All PROs showed statistically significant improvements starting at 6 months and maintained through 5‐year. Pre‐operative standing full‐length x‐rays, averaged 6.5° varus. The average change in mechanical axis was less than 1° at 5‐year. No difference in PROs was found between patients with (*N* = 63) and without (*N* = 27) radiolucencies. Only two cases with radiolucencies progressed to revision. There was one superficial infection and four revisions to the total knee arthroplasty. No thromboembolic, instability or arthrofibrosis complications occurred. Kaplan–Meier survivorship was 98.5% at 2‐year and 98.1% at 5‐year.

**Conclusion:**

Unicompartmental knee replacement with implant system that allows surgical flexibility and independent balancing is a surgical option with promise of excellent functional outcomes, low complication rates and high survivorship (98.1% at 5‐year). These results challenge previous studies indicating high revision rates and support the utilisation of unicompartmental arthroplasty for properly indicated patients.

**Level of Evidence:**

Level II, prognostic studies.

**Abbreviations**
ACL: anterior cruciate ligament
ADL: activities of daily living
AE(s): adverse event(s)
ANOVA: analysis of variance
AP: anteroposterior
BMI: body mass index
DVT: deep vein thrombosis
FJS: forgotten joint score
IRB: Institutional Review Board
KL: Kellgren–Lawrence
KOOS: knee injury and osteoarthritis outcome score
MCL: medial collateral ligament
OKS: Oxford knee score
PA: posteroanterior
QOL: quality of life
ROM: range of motion
TKA: total knee arthroplasty
UKA: unicompartmental knee arthroplasty
VAS: visual analogue scale
X‐rays: radiographic images

## INTRODUCTION

Knee arthritis is a major source of suffering and disability worldwide [48]. A combination of improved longevity, increased activity levels in aging adults, and the demographics of an aging population all point to a dramatic increase in the need for knee arthroplasty over time. While total knee arthroplasty has a long history of improved outcomes, up to 20% of patients report dissatisfaction [13]. There are many proposed explanations for this, including instability, arthrofibrosis, infection, prosthetic design, loss of proprioception and altered knee kinematics due to ligament resection [23].

Unicompartmental knee arthroplasty (UKA) is a viable option for unicompartmental knee osteoarthritis, and, in properly selected cases, has been becoming increasingly popular. Several advantages to UKA have been proposed including: less tissue dissection, less operative time, preservation of cruciate ligaments, faster recovery, fewer complications and better patient‐reported outcomes [43]. The most important feature of UKA is a significant reduction in perioperative risks [46]. While these short‐term advantages have been used to justify the use of UKA for properly indicated patients, historically, the criticisms have been with the long‐term durability and survivorship. Older studies cite higher revision rate primarily due to loosening and opposite compartment progression and subsequent failure [34].

Newer studies have seen better survivorship [29], much due to improvement in patient selection and surgical technique with increased appreciation in avoiding alignment overcorrection. Advancements in implants and instrumentation have resulted in compartment‐specific implants, precise implant sizing options, improved instrumentation accuracy and appreciation of proper gap balancing. Greater surgeon utilisation has contributed to improvements in implant survivorship and drastically reduced revision rates [9, 21].

In line with this more recent data, the purpose of this study was to report the midterm patient‐reported outcomes and survivorship of the Medacta MOTO Medial® Unicompartmental system (Medacta International). The surgical procedure focuses on implantation using a balanced, aligned resection technique to minimise change to the subjects' preoperative knee geometry and alignment [45].

## MATERIALS & METHODS

This was a prospective, multicenter study, designed to assess mid‐term performance of the Medacta MOTO Medial® Unicompartmental prosthesis (*Medacta International, Castel San Pietro, Switzerland)*, with patient‐reported outcomes, clinical findings and radiographic analysis. Data was included from four United States based sites. The study's protocol was designed to collect data at the following time points: pre‐operative, intraoperative, 4 weeks, 6 months, 1‐year, 2‐year and 5‐year. Outcomes at each time point included: radiographic images, adverse events and clinical assessment. Patient‐reported outcome scores collected include the Oxford knee score (OKS), forgotten joint score (FJS), visual analogue scale (VAS) and knee injury and osteoarthritis outcome score (KOOS). All investigating surgeons have 15–30 years' experience in knee arthroplasty and partial knee replacement. Institutional Review Board (IRB) approval was obtained for the study.

Data is reported on 207 participants implanted across the four sites between October 2017 and June 2022. Of the 207 participants, 23 participants have withdrawn their consent, 11 participants have been lost to follow‐up and 3 participants have died unrelated to the study. A total of 170 participants reached the 2‐year follow‐up and 90 have reached the 5‐year follow‐up.

Participants' ages who were implanted ranged from 43 to 85 years old with a mean age of 64.7 [16, 17]. 52% were male and 48% were female. The average body mass index (BMI) recorded was 33. Laterality of procedure was 52% right knee and 48% left knee. The majority, 204 participants, were diagnosed with osteoarthritis, 3 participants were diagnosed with osteochondritis dissecans lesions and secondary osteoarthritis, 2 of which suffered medial condyle collapse.

**Figure 1:**
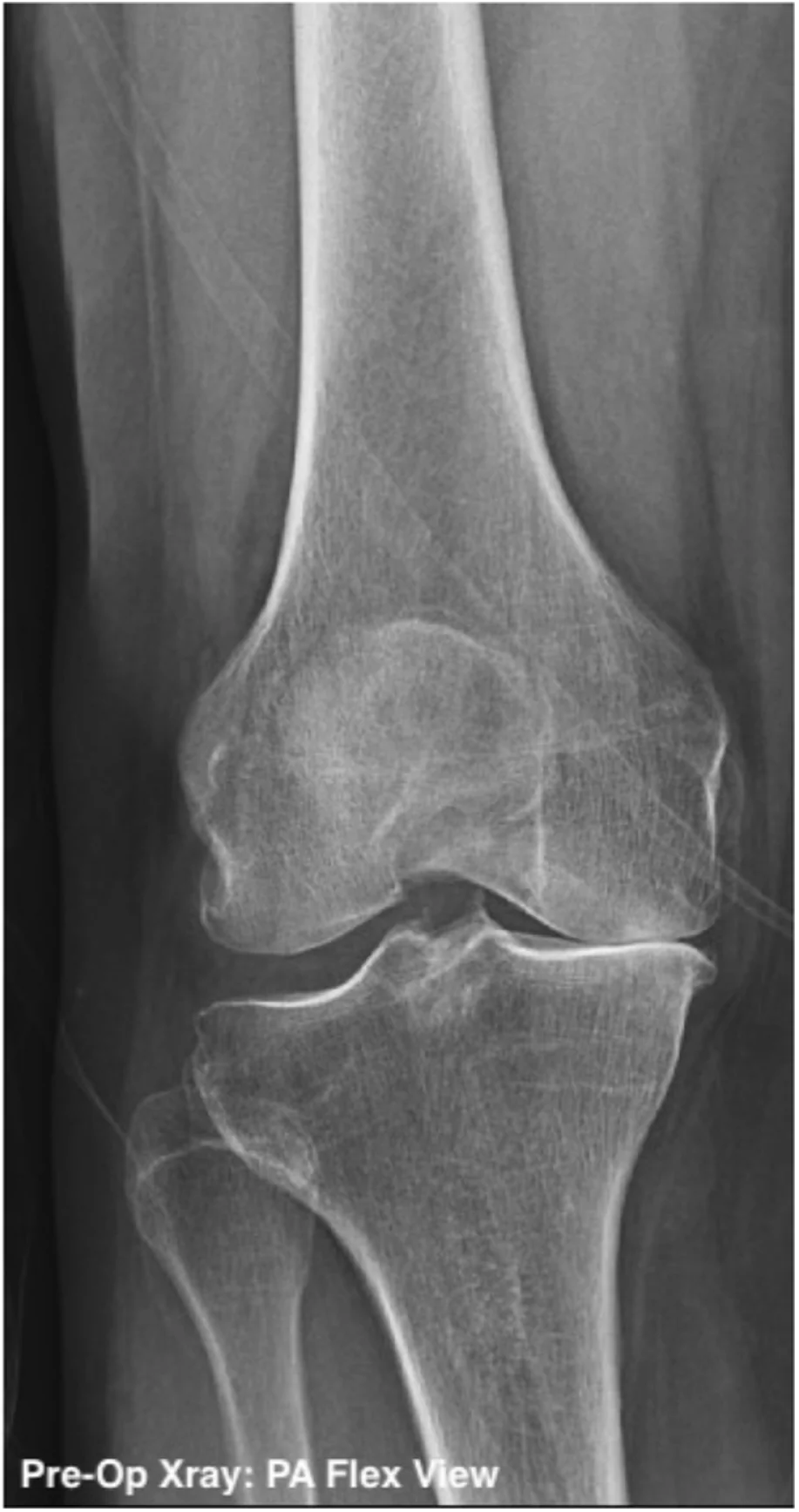
Pre‐op x‐ray: PA flex view. PA, posteroanterior.

**Figure 2:**
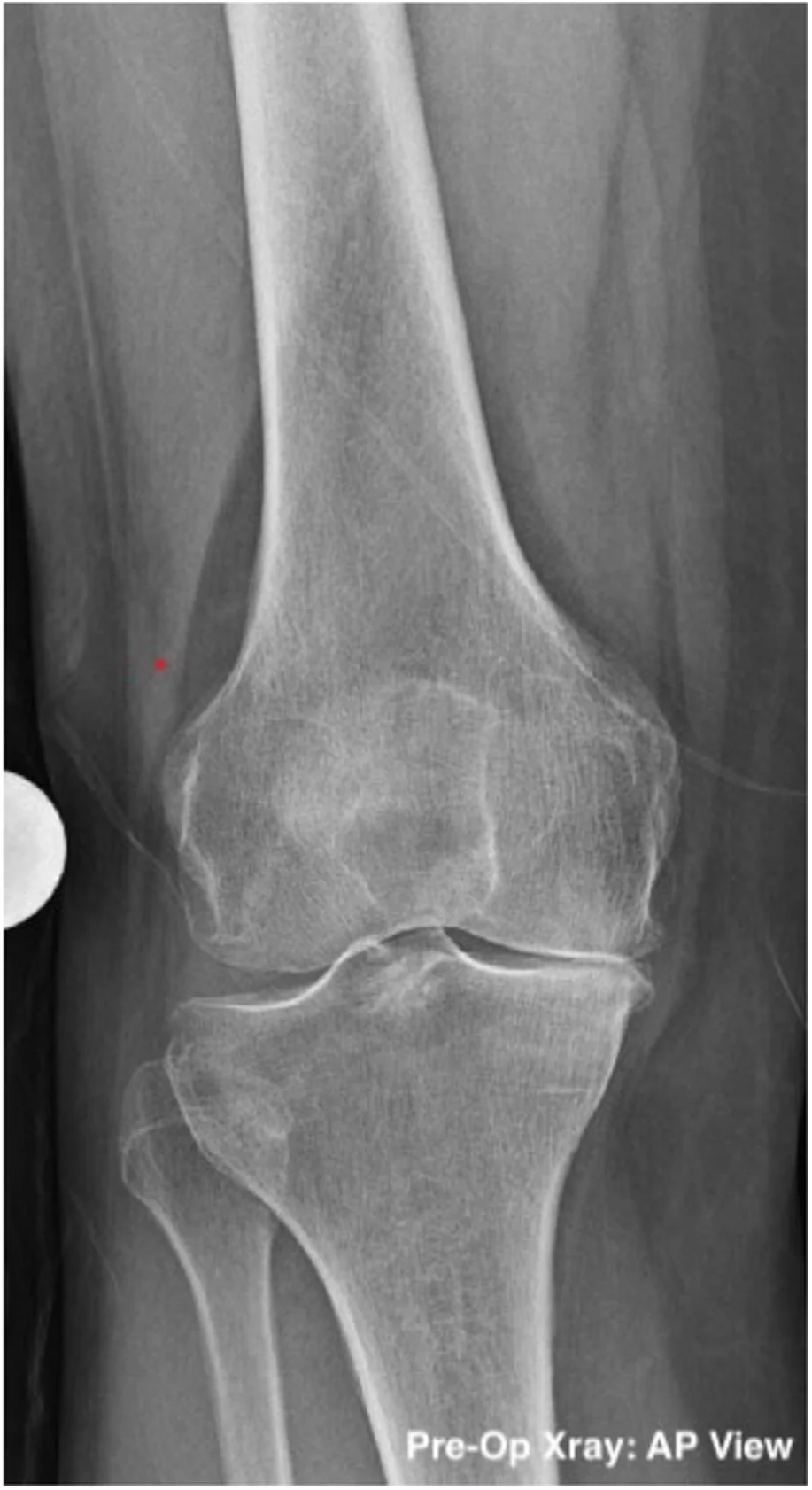
Pre‐op x‐ray: AP view. AP, anteroposterior.

**Figure 3:**
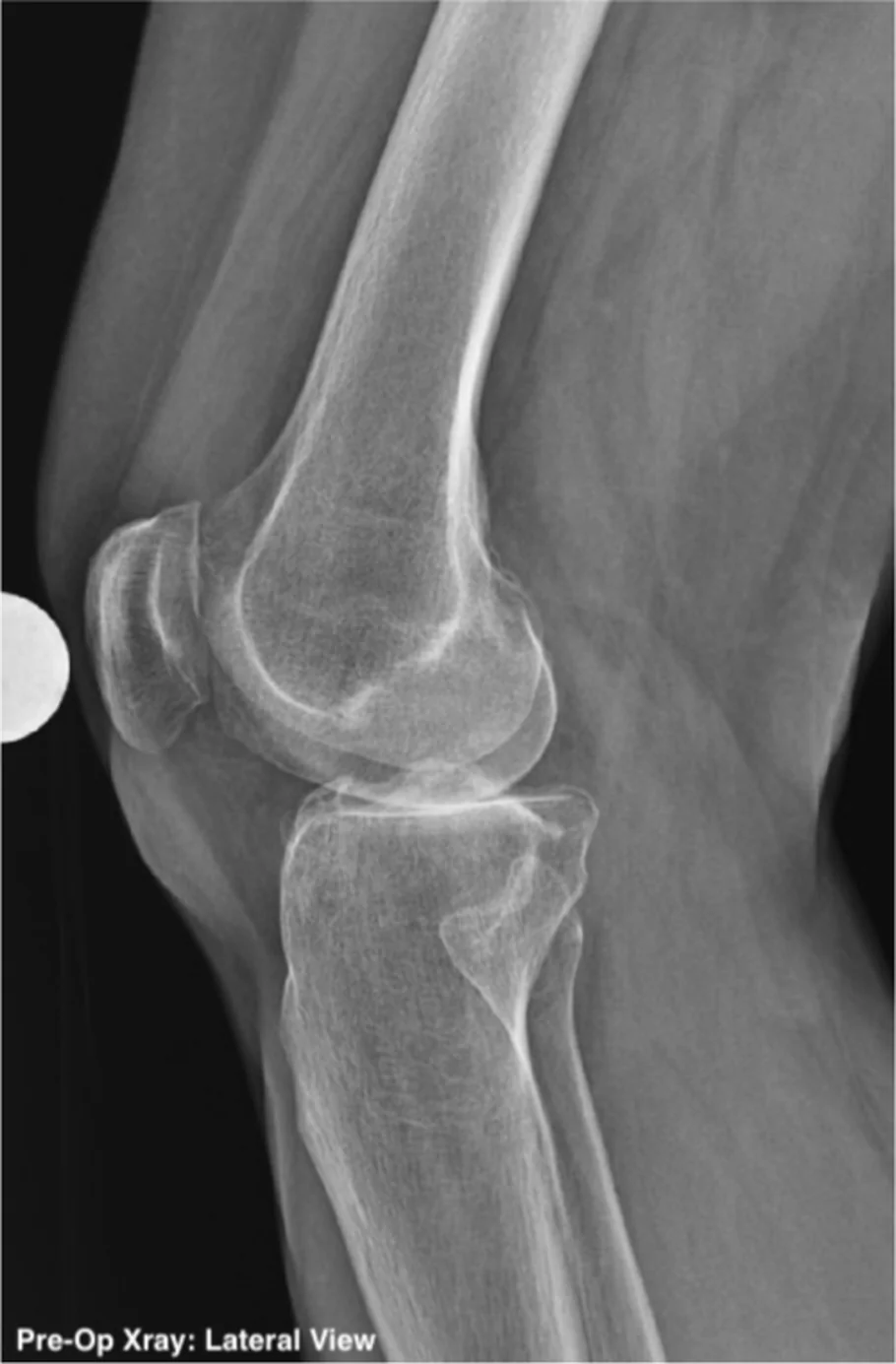
Pre‐op x‐ray: Lateral view.

**Figure 4:**
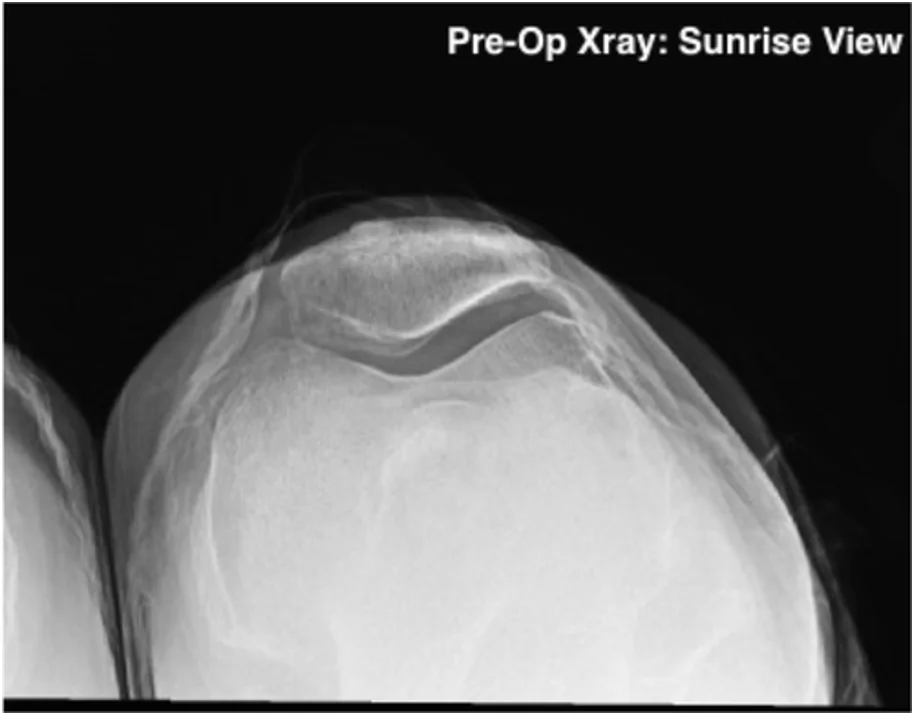
Pre‐op x‐ray: Sunrise view.

Preoperative history, examination and radiographs were performed to determine appropriateness for medial UKA. No patient restrictions were made on BMI, age or activity level [32]. Exams included, but were not limited to, range of motion, stability, location of pain and correctability of the varus deformity. A range of motion greater than flexion of 100 degrees with flexion contracture less than 10 degrees was acceptable. Stability with cruciates and collaterals were required to be intact. Location of pain was preferred to be largely isolated to the medial compartment although some anterior knee pain was allowed provided radiographs confirmed adequate patellofemoral joint space. Amount of varus deformity was variable, but preferred to be correctable or near correctable on exam and stress x‐ray. Lateral compartment integrity was evaluated clinically and radiographically with valgus stress x‐rays, with a requirement of normal cartilage and joint space. Stress x‐rays were divided into three types, Type I when the medial compartment joint space opens less than the lateral compartment joint space by greater than 1 mm, Type II with the medial and lateral compartment joint space within 1 mm and Type III with the medial joint space opening greater than 1 mm compared to the lateral compartment. Other standard radiographs included anterior/posterior, lateral, flexion PA, sunrise and long leg hip to ankle (Figures 1, 2, 3, 4). Joint spaces were digitally calculated and Kellgren–Lawrence (KL) grading [36] performed for all compartments. Mechanical and anatomic axis were recorded from the standing long leg radiographs. See Table 1 for full Inclusion and Exclusion Criteria

**Table 1:** Inclusion and exclusion criteria.

Inclusion criteria	Exclusion criteria
Standard of care criteria to implant a medial unicompartmental device Must be able to read, understand and provide written informed consent on the Institutional Review Board (IRB) approved Informed Consent Form (ICF). Ability to understand and provide written authorisation for use and disclosure of personal health information. Subjects who are able and willing to comply with the study protocol and follow‐up visits. Must be 18 years or older to participate. Subjects must have medial knee disease in the affected knee compliant with the U.S. Food & Drug Administration‐approved indications for use of medial UKA. Must have had no prior arthroplasty to the medial compartment of the study knee. Patients who are candidates for a unicompartmental knee arthroplasty and are determined to undergo a UKA will be offered enrolment. Subjects must be able to return for the follow‐up appointments and have the mental capacity to cooperate and complete PRO questionnaires, physical exam and radiographs.	Knee ligament instability (deficiency of cruciate or collateral ligaments) Inflammatory arthritis History of prior knee infection History of alcoholism or drug abuse Currently on chemotherapy or radiation therapy for neoplastic disease. Currently on immunosuppressive medications including systemic steroids History of known sensitivity or allergy to materials used in orthopaedic implants, specifically Titanium and Cobalt‐Chrome alloys Habitual use of narcotic pain medications prior to surgery (more than three doses or pills per week) History of metabolic disorder affecting the skeletal system other than osteoarthritis or osteoporosis (e.g., Osteomalacia, Ricketts) History of chronic pain issues for reasons other than knee pain. Women who are pregnant. Unstable psychiatric illness

Abbreviations: PRO, patient‐reported outcome; UKA, unicompartmental knee arthroplasty.

Surgical facility and postoperative disposition were at the investigators' discretion. All surgeries received preoperative antibiotics, tranexamic acid [39] and were performed in the investigators standard fashion without navigation or robotic assistance. Surgeries were performed through a medial arthrotomy. The surgical technique is a tibia‐first resection. Proximal tibia resection slope and coronal plane alignment were to follow the patient's anatomy through an extramedullary guide. Gap balancing was performed in flexion and extension with an emphasis on avoiding overcorrection with repetitive alignment checks. Tibia recuts were made as needed, with a goal of 0–3 degrees varus and slope of 3–7 degrees. The distal femoral cut was linked to the tibia cut with an extension block and guide. The distal femoral resection was completed to obtain an extension gap of 15 mm, while maintaining varus knee position. Femur was sized and finished. The posterior femoral cut was made accounting for the typical intact posterior cartilage, usually requiring an additional two millimetres to the resection. The largest tibial baseplate possible without medial overhang was selected. A few millimetres of anterior overhang was accepted, no more than 2–3 mm. Efforts were made to ensure centreing of the femoral component on the tibia component centre point in both flexion and extension. Trialling was performed to determine the best polyethylene thickness. All components were cemented. Antibiotic cement utilisation was at the surgeon's discretion after patient risk stratification.

**Figure 5:**
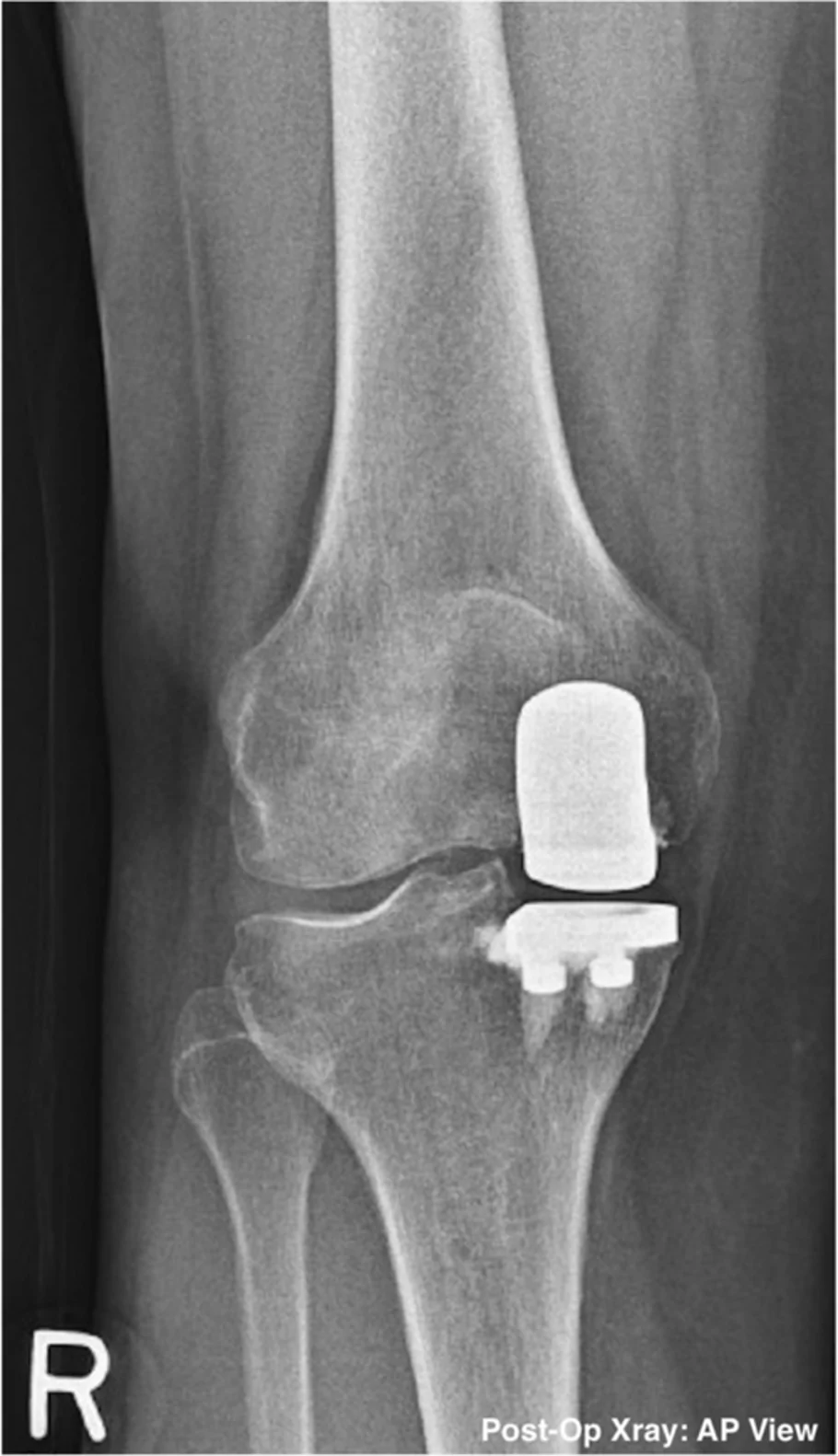
Post‐op x‐ray: AP view. AP, anteroposterior.

**Figure 6:**
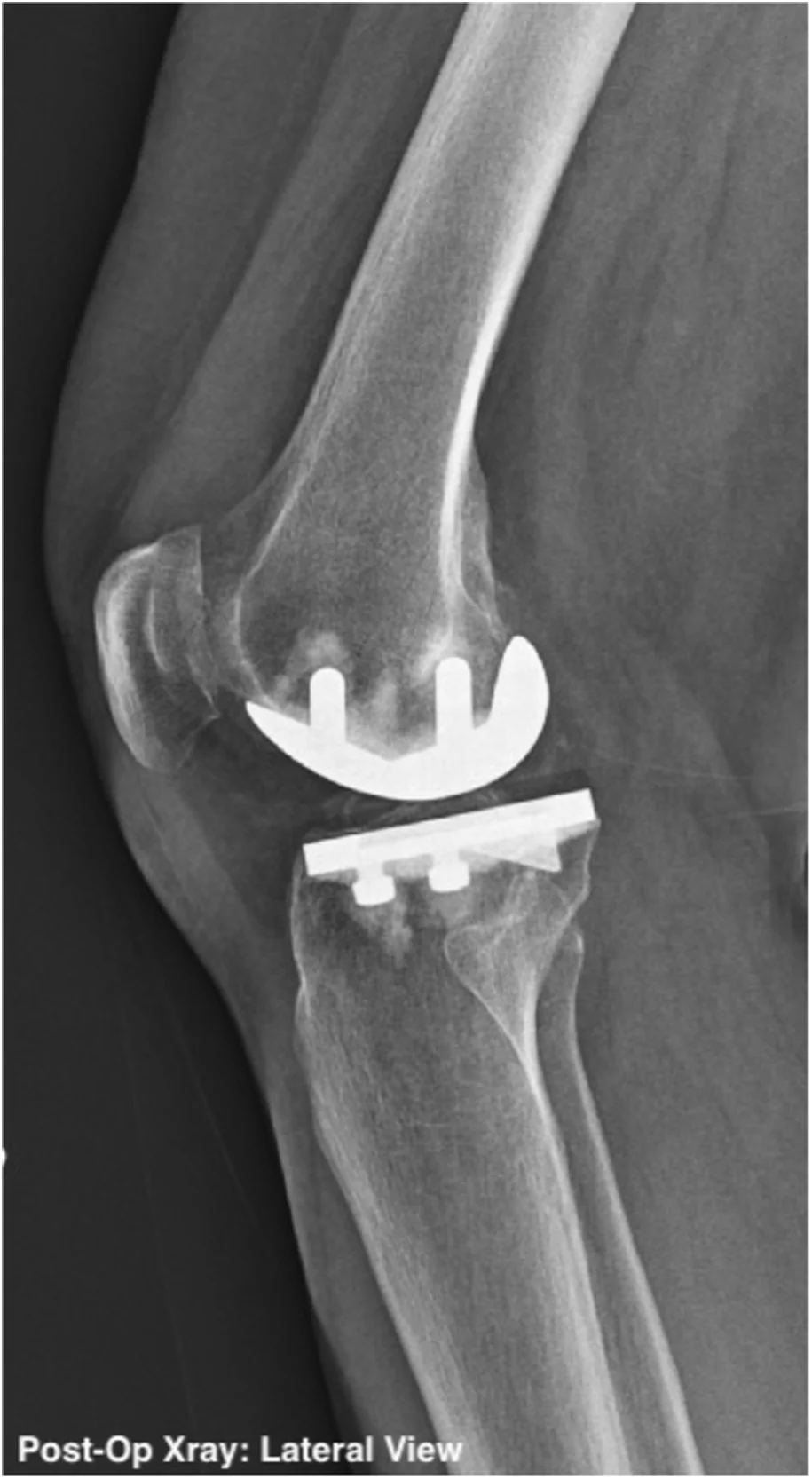
Post‐op x‐ray: Lateral view.

**Figure 7:**
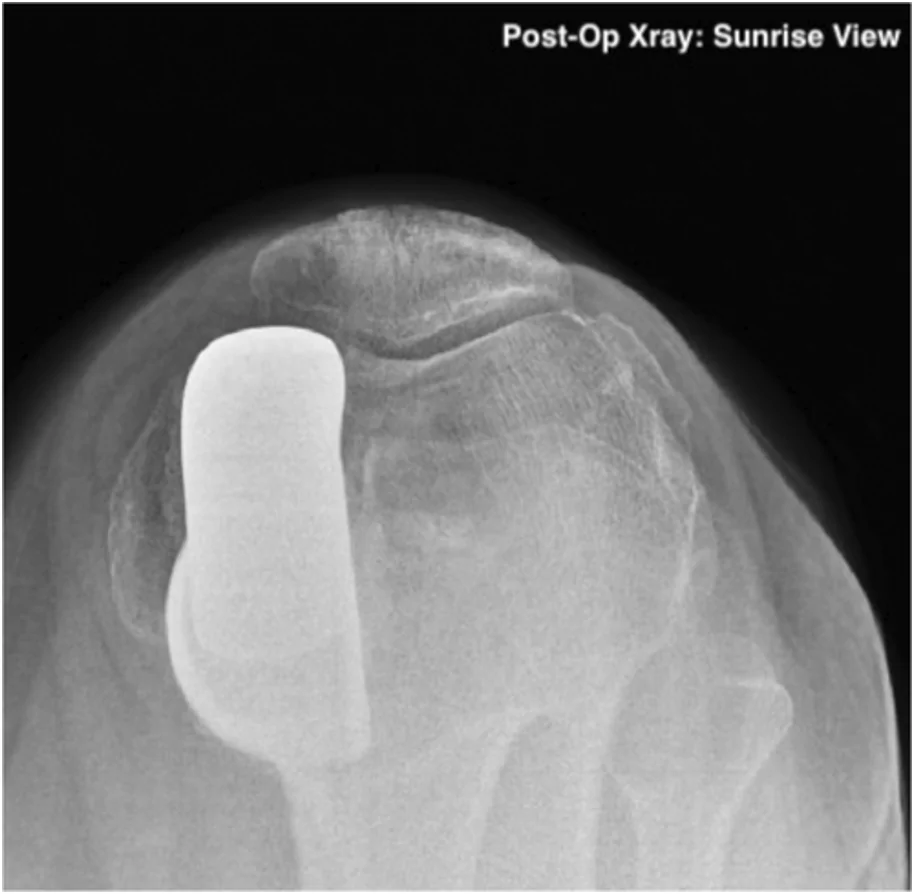
Post‐op x‐ray: Sunrise view.

**Figure 8:**
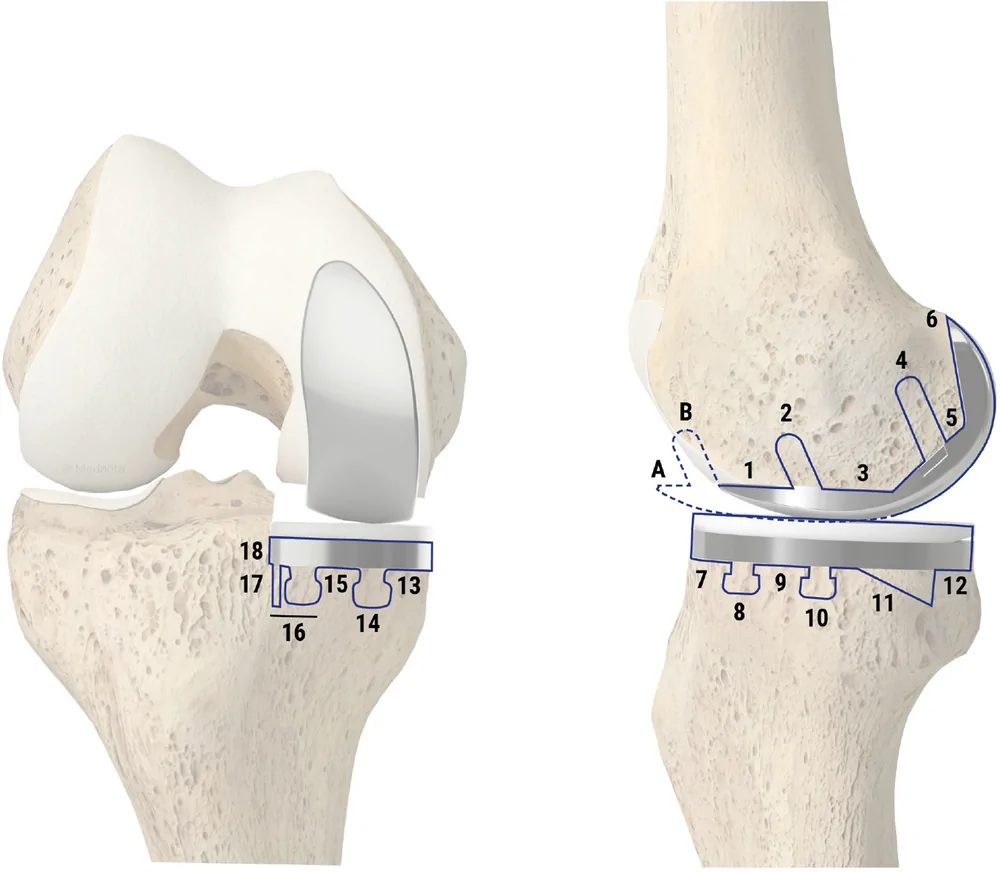
Radiolucent line zones.

Follow‐up visits included examinations, radiographs and patient‐reported outcome scores. Postoperative radiographs AP, lateral, sunrise, pa flexion and standing long leg were evaluated for nonresurfaced compartment progression, implant position, radiolucencies and implant loosening (Figures 5, 6, 7). Radiolucencies were digitally measured through twelve of tibial zones and eight femoral zones and any progression noted into adjacent zones (Figure 8). A Kaplan–Meier survivorship analysis was performed at 2 and 5 years with revision for any cause. The four revisions were analysed for cause with review of the operative note and radiographs. Multivariable logistic regression models were used to assess factors associated with achieving the minimally clinical importance difference (MCID) and patient acceptable symptom state (PASS) thresholds, adjusting for age, BMI and centre/surgeon effect. A conservative worst‐case sensitivity analysis was performed at the 5‐year follow‐up, classifying patients lost to follow‐up as nonresponders.

## RESULTS

### Radiographs

**Figure 9:**
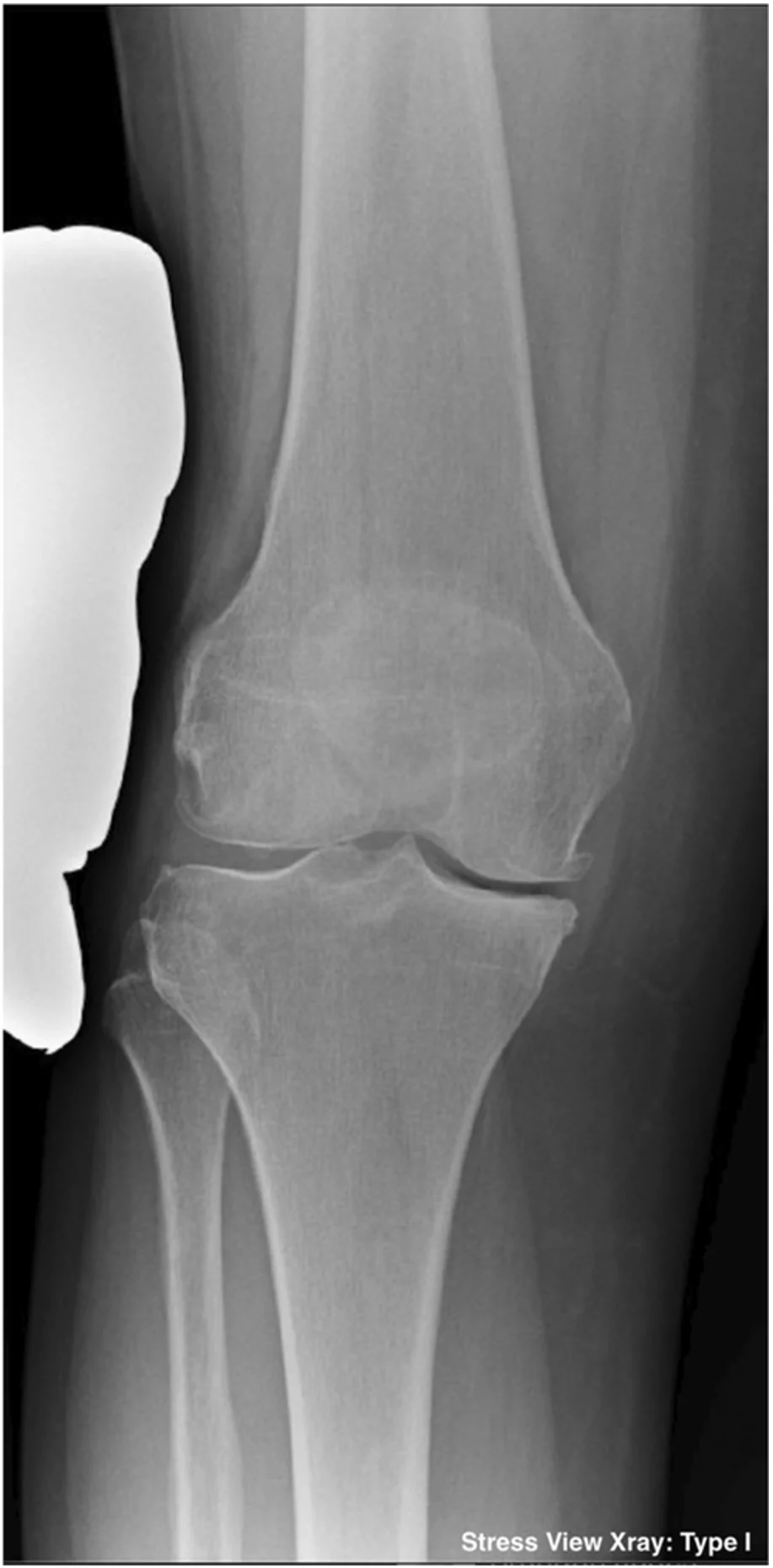
Stress view x‐ray: Type I.

**Figure 10:**
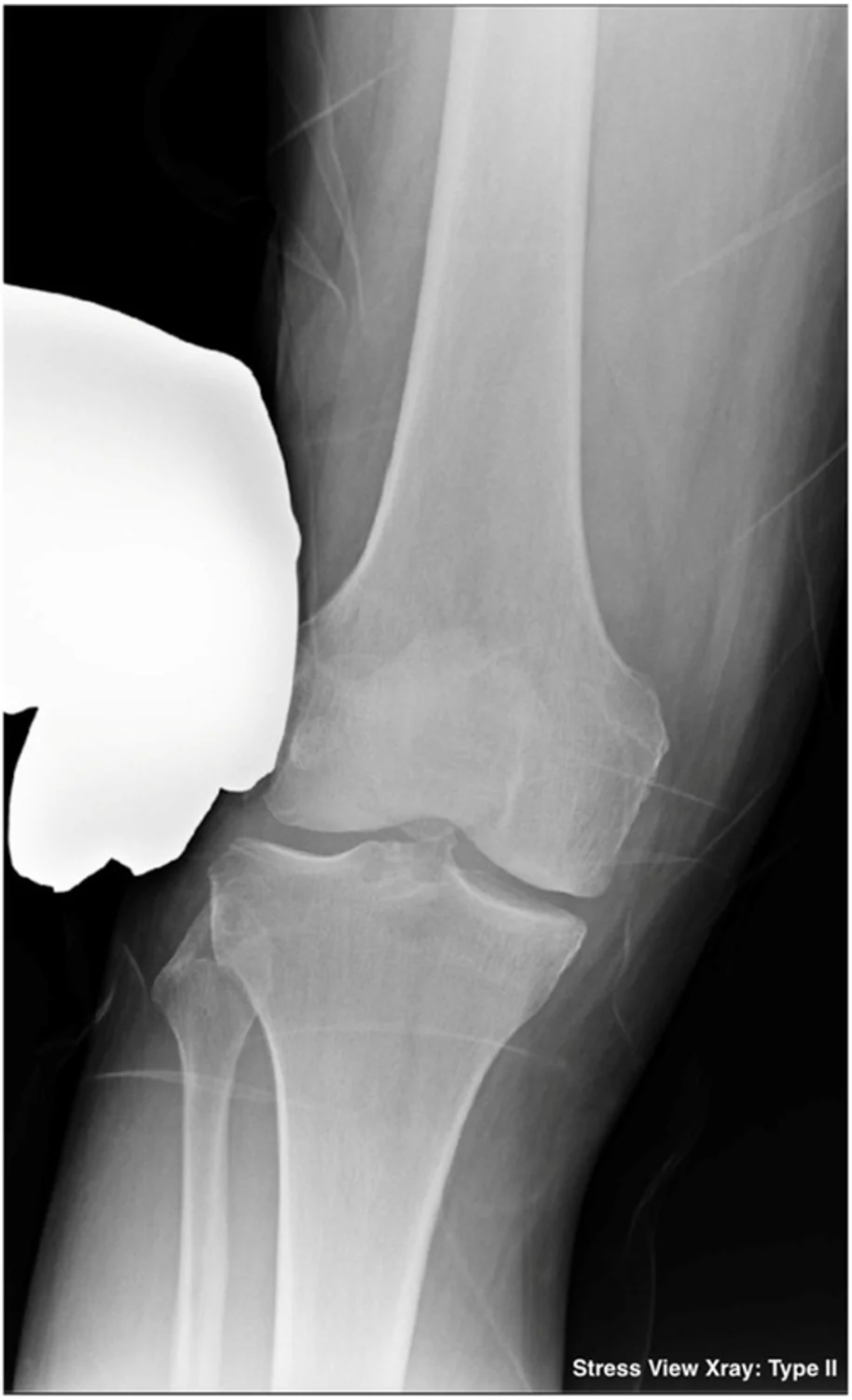
Stress view x‐ray: Type II.

**Figure 11:**
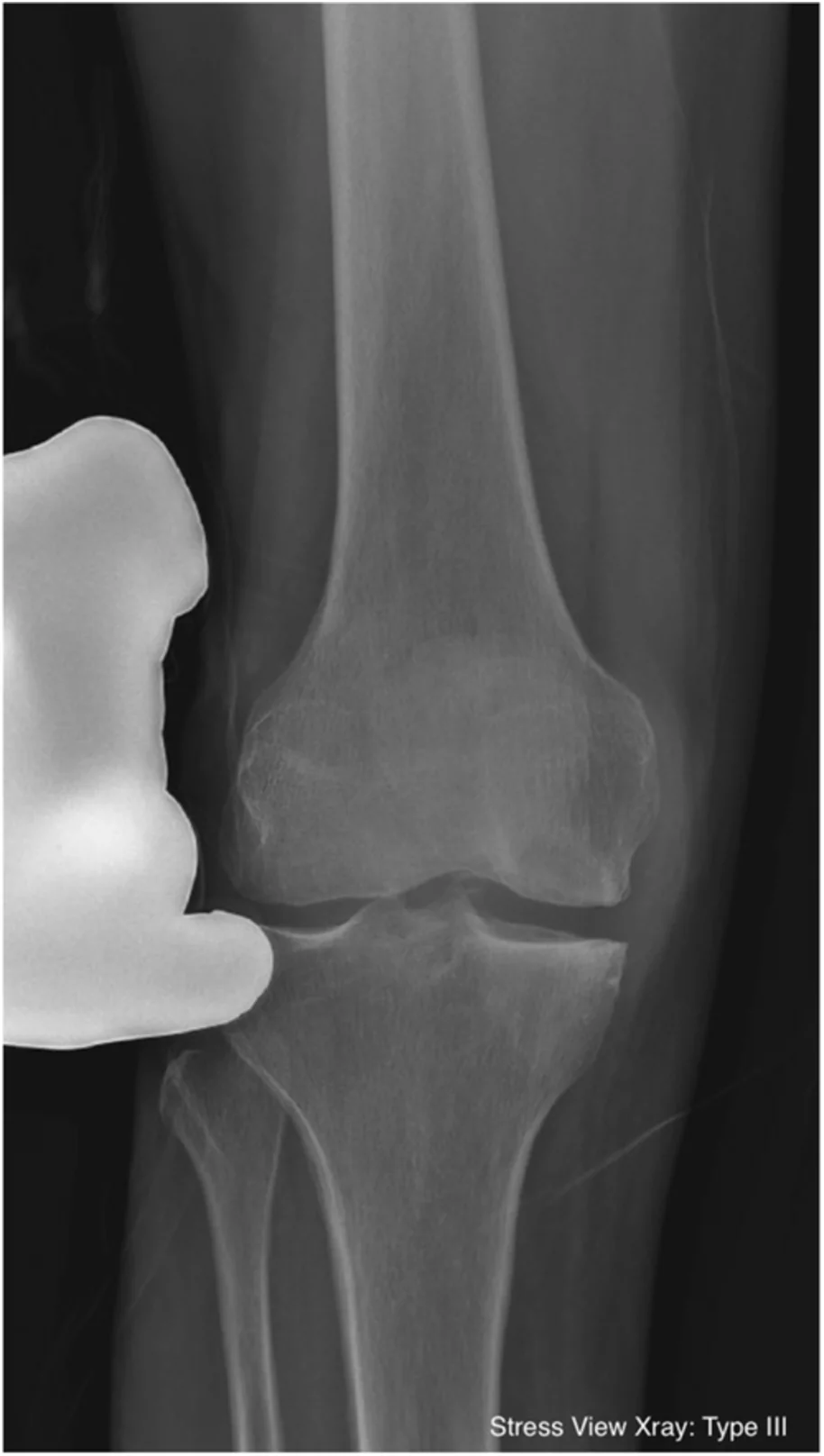
Stress view x‐ray: Type III.

Preoperative radiographs demonstrated the majority of patients 96% with Kellgren Lawrence (KL) grade 3–4 medial compartment arthritis (Table 2). The remaining cases were osteochondritis dissecans with medial condyle collapse. Patellofemoral KL arthritic changes were predominantly grade 2 or less, although 4.8% were grade 3–4 (Table 2). Participants with a patellofemoral KL arthritic Grade of 3–4 had outcomes that aligned with the remaining cohort's average. None of the four revisions occurred in the patellofemoral KL Grade 3–4 group. Lateral compartment grading was 0–1 for 98.5% of patients (Table 2). This study evaluated valgus stress radiographs and divided these into the amount of opening of the medial compartment relative to the lateral compartment joint space (Figures 9, 10, 11). The vast majority of cases were Type I (30.1%) and Type II (53.4%). Knees that showed potential to open into a more valgus position, the Type III stress x‐ray, were less frequently seen, only 16.5%. Further analysis of the Type III stress x‐ray found 41% started with a mechanical axis at slight varus to neutral (Table 3). Of the cases that survived to 5‐year the average change in the mechanical axis from preoperative was less than 1 degree (Table 4). Preoperative native alignment on standing full‐length x‐rays averaged 6.5 degrees varus. The 5‐year mechanical axis averaged 5.8 degrees varus (Table 5).

**Table 2:** Rosenberg K&L grade & Kellgren–Lawrence grade.

	Rosenberg medial compartment KL grade	Rosenberg lateral compartment KL grade	PF Joint KL grade
Count	206	206	206
None 0	0 (0.0%)	142 (68.9%)	63 (30.6%)
Grade 1	1 (0.5%)	62 (30.1%)	89 (43.2%)
Grade 2	7 (3.4%)	2 (1.0%)	44 (21.4%)
Grade 3	52 (25.2%)	0 (0.0%)	7 (3.4%)
Grade 4	146 (70.9%)	0 (0.0%)	3 (1.4%)

**Table 3:** Stress view x‐rays & stress view type III measurement breakdown.

Stress view x‐rays			Type III measurement breakdown	
Count	206	%	165–169	2
Type I	62	30.1%	170–174	18
Type II	110 (3 revisions)	53.4%	175–179	11
Type III	34 (1 Revision)	16.5%	180–184	1
			185–189	2

**Table 4:** Long leg x‐rays—mechanical axis change per individual participant.

	6 months versus Pre‐op	2 years versus Pre‐op	5 years versus Pre‐op	5 years versus 2 years	2 years versus 1 year	2 years versus 6 months	5 years versus 6 months
Count	193	155	88	81	139	152	88
Average	1.9	1.3	0.5	−0.1	1.2	0.8	−0.7
Min	−16.3	−14.5	−8.6	−3.3	−5.0	−7.9	−9.8
Max	11.6	13.1	11.9	5.2	11.2	10.3	4.5

**Table 5:** Long leg x‐rays—mechanical axis.

	Pre‐op	6 months	1 year	2 years	5 years
Count	206	193	161	156	89
Avg	173.5	175.4	175.0	175.0	174.2
Median	173.5	175.5	175.0	175.0	173.8
SD	4.3	3.2	3.2	3.4	3.8
Min	144.7	167.2	167.1	166.8	165.7
Max	189.6	185.7	182.7	184.7	182.8

Abbreviation: SD, standard deviation.

### Radiolucencies

Radiolucencies (RLLs) were measured at 6 month, 1‐year, 2‐year and 5‐year follow‐up. The findings included 50 patients with radiolucencies and 63 patients, who have completed the study, without any radiolucent lines. The noted zones of lucency of the tibial component were most commonly seen at the medial‐most aspect of the tibial baseplate and the lateral border of the sagittal cut. Femoral component radiolucent lines were most often seen along the posterior cut (Table 6). Progression was seen in two cases that were revised for tibial component loosening. The rest of the cases were nonprogressive radiolucent lines. In a preliminary analysis, outcomes at 5 years were compared between 27 patients with radiolucent lines and 63 patients without radiolucent lines. With the exception of the FJS favoring the radiolucent line group (average 77.8, median 85.4) over the no line group (average 72.2, median 81.3), no other significant differences were found for either group in any of the other patient reported outcomes (Tables 7, 8, 9). This preliminary data will be thoroughly explored in future analysis and manuscripts. No changes in implant position were seen at 5‐year follow‐up.

**Table 6:** Radiolucent lines.

	6 months	1 year	2 years	5 years
No RLLs	178^a^	158	136	76
RLLs	14	27	22	13
RLL zones	6, 7, 11, 12, 13, 14, 17, 18	A, B, 1, 3, 5, 6, 7, 8, 10, 12, 13, 14, 15, 16, 17, 18	B, 1, 5, 6, 7, 8, 9, 10, 11, 12, 13, 14, 15, 16, 17, 18	1, 3, 7, 11, 12, 13, 15, 18
Revisions	1^b^	2^c^	1^c^	0
Missed RGs	4	2	9	1
Missed visit	5	7	9	0
Pending visit	0	0	0	74

^a^ 1 revision case did not have any RLLs at 6 months. The revision occurred after the 6‐month visit due to advancing osteoarthritis.

^b^ 1 revision case had progressive RLLs at 6 months and 1 year. The revision occurred after the 1‐year visit due to aseptic loosening.

^c^ 1 revision case had no RLLs present at 6mo but reported progressive RLLs at 1 year and 2 years. The revision occurred after the 2‐year visit due to aseptic loosening.

**Table 7:** OKS with RLLs present compared to OKS without RLLs present.

OKS with RLLs present						OKS without RLLs present					
	Pre‐op	6 months	1 year	2 years	5 years		Pre‐op	6 months	1 year	2 years	5 years
Count	48	44	44	44	27	Count	150	137	129	123	63
Average	21.9	42.5	43.2	43.6	44.2	Average	23.3	41.5	43.4	43.7	43.3
Median	21.5	43.0	46.0	46.0	46.0	Median	24.0	43.0	45.0	46.0	45.0
SD	6.1	5.0	6.9	6.1	5.5	SD	6.6	6.1	5.4	4.6	4.7
Min	11	26	18	23	28	Min	4	19	23	29	25
Max	37	48	48	48	48	Max	37	48	48	48	48

Abbreviations: OKS, Oxford knee score; SD, standard deviation.

**Table 8:** FJS with RLLs present compared to FJS without RLLs present.

FJS with RLLs present					FJS without RLLs present				
	6 months	1 year	2 years	5 years		6 months	1 year	2 years	5 years
Count	44	45	44	27	Count	135	126	124	63
Average	68.6	73.8	80.5	77.8	Average	64.5	75.3	76.4	72.2
Median	76.0	87.5	88.5	85.4	Median	72.9	82.3	87.5	81.3
SD	29.2	29.1	23.1	25.8	SD	29.7	24.2	24.8	28.9
Min	0	0	20.8	14.6	Min	0	4.2	6.3	0
MaxA	100	100	100	100	Max	100	100	100	100

Abbreviations: FJS, forgotten joint score; SD, standard deviation.

**Table 9:** KOOS with RLLs present compared to KOOS without RLLs present.

KOOS with RLLs present											
Pain						Symptoms					
	Pre‐op	6 months	1 year	2 years	5 years		Pre‐op	6 months	1 year	2 years	5 years
Count	48	44	46	44	27	Count	48	44	46	44	27
Average	46.1	89.1	89.1	91.3	93.0	Average	49.3	81.7	85.0	89.9	89.7
Median	47.2	93.1	97.2	97.2	97.2	Median	50.0	83.9	95.6	92.9	92.9
SD	12.1	12.6	17.2	13.0	9.9	SD	14.3	15.2	16.8	12.5	11.2
Min	22.2	52.8	36.1	44.4	69.4	Min	14.3	39.3	21.4	42.9	64.3
Max	86.1	100.0	100.0	100.0	100.0	Max	78.6	100.0	100.0	100.0	100.0

ADL						Sport					
	Pre‐op	6 months	1 year	2 years	5 years		Pre‐op	6 months	1 year	2 years	5 years
Count	48	44	46	44	27	Count	48	44	46	44	27
Average	49.8	90.0	89.2	92.9	93.5	Average	20.9	67.2	73.7	72.5	80.9
Median	46.3	94.1	95.6	97.1	97.1	Median	20.0	75.0	80.0	80.0	90.0
SD	14.4	11.2	16.8	12.2	9.3	SD	18.0	28.0	24.7	25.5	21.4
Min	13.2	50.0	26.5	48.5	66.2	Min	0.0	0.0	5.0	5.0	40.0
Max	91.2	100.0	100.0	100.0	100.0	Max	75.0	100.0	100.0	100.0	100.0

QOL											
	Pre‐op	6 months	1 year	2 years	5 years						
Count	48	44	46	44	27						
Average	21.1	78.7	81.3	81.7	85.6						
Median	18.8	84.4	87.5	87.5	93.8						
SD	15.7	18.5	20.5	21.2	19.3						
Min	0.0	25.0	12.5	31.3	37.5						
Max	56.3	100.0	100.0	100.0	100.0						

KOOS without RLLs present											
Pain						Symptoms					
	Pre‐op	6 months	1 year	2 years	5 year		Pre‐op	6 months	1 year	2 year	5 years
Count	149	134	129	124	63	Count	149	134	129	124	63
Average	48.1	88.5	91.4	91.5	92.0	Average	54.3	84.8	88.6	89.9	88.9
Median	50.0	94.4	97.2	97.2	97.2	Median	53.6	89.3	92.9	92.9	92.9
SD	15.3	13.7	11.6	12.2	10.4	SD	16.5	13.6	11.1	12.5	10.9
Min	2.8	30.6	47.2	38.9	53.0	Min	0.0	39.3	50.0	53.6	50.0
Max	83.3	100.0	100.0	100.0	100.0	Max	93.0	100.0	100.0	100.0	100.0

ADL						Sport					
	Pre‐op	6 months	1 year	2 years	5 years		Pre‐op	6 months	1 year	2 years	5 years
Count	149	134	129	124	63	Count	149	134	129	124	63
Average	51.3	88.9	92.1	92.9	90.8	Average	18.8	59.5	70.3	72.5	73.2
Median	50.0	94.1	95.6	97.1	97.1	Median	15.0	62.5	75.0	80.0	80.0
SD	15.8	13.0	10.2	12.2	11.6	SD	18.8	29.9	27.8	25.5	25.9
Min	0.0	30.9	47.1	48.5	52.9	Min	0.0	0.0	0.0	10.0	5.0
Max	92.9	100.0	100.0	100.0	100.0	Max	89.7	100.0	100.0	100.0	100.0

QOL											
	Pre‐op	6 months	1 year	2 years	5 years						
Count	149	134	129	124	63						
Average	23.1	74.7	81.8	81.7	81.6						
Median	18.8	75.0	87.5	87.5	87.5						
SD	17.7	20.6	19.0	21.2	20.4						
Min	0.0	12.5	18.8	18.8	18.8						
Max	81.3	100.0	100.0	100.0	100.0						

Abbreviations: ADL, activities of daily living; KOOS, knee injury and osteoarthritis outcome score; QOL, quality of life; SD, standard deviation.

### Patient‐reported outcomes (PROs)

All PROs demonstrated statistically significant improvements, as revealed by repeated measures ANOVA (*p* < 0.001) (Table 10). The FJS increased significantly from 6 months postoperatively (mean: 65.1, median: 72.9) to 1 year (mean: 75.0, median: 85.4) and 2 years (mean: 77.2, median: 87.5). An insignificant nominal decrease was observed at 5 years (mean: 74.0, median: 83.3), but the score remained significantly higher than at 6 months (*p* < 0.05) (Table 11). The OKS showed substantial and statistically significant improvements from preoperative values to all postoperative follow‐up visits (*p* < 0.001), with continued improvement observed from 6 months onward. KOOS pain, symptoms and activities of daily living (ADL) subscales also demonstrated significant postoperative improvements (*p* < 0.001), which stabilised after the 6‐month follow‐up. Similarly, KOOS Sports and quality of life (QOL) subscales showed significant gains after surgery (*p* < 0.001), continuing through the first year of follow‐up and stabilising thereafter through the 5‐year follow‐up (*p* < 0.05). Post hoc comparisons between time points were performed using the Tukey pairwise test (Tables 12, 13, 14, 15).

**Table 10:** Summary of statistical analysis.

Score	ANOVA *p*‐value	Significant difference
FJS	< 0.001	6 months versus 1/2/5 years
OXFORD	<0.001	Pre‐op versus others
		6 months versus 1/2/5 years
KOOS‐pain	<0.001	Pre‐op versus others
KOOS‐Symp.	<0.001	Pre‐op versus others
		6 months versus 1/2/5 years
KOOS‐ADL	<0.001	Pre‐op versus others
KOOS‐Sport	<0.001	Pre‐op versus others
		6 months versus 1/2/5 years
KOOS‐QOL	<0.001	Pre‐op versus others
		6 months versus 1/2/5 years

Abbreviations: ADL, activities of daily living; ANOVA, analysis of variance; FJS, forgotten joint score; KOOS, knee injury and osteoarthritis outcome score; QOL, quality of life.

**Table 11:** FJS with revision cases and FJS without revision cases.

FJS with revision cases					FJS without revision cases				
	6 months	1 year	2 years	5 years		6 months	1 year	2 years	5 years
Count	182	176	170	90	Count	179	174	169	90
Average	65.1	75.0	77.2	74.0	Average	65.4	75.2	77.5	74.0
Median	72.9	85.4	87.5	83.3	Median	72.9	85.4	87.5	83.3
SD	29.5	25.8	24.5	27.9	SD	29.5	25.4	24.3	27.9
Min	0	0	6	0	Min	0	0	6	0
Max	100	100	100	100	Max	100	100	100	100

Abbreviations: FJS, forgotten joint score; SD, standard deviation.

**Table 12:** Forgotten joint score Tukey pairwise comparisons: Visit grouping information using the Tukey method and 95% confidence.

Visit	*N*	Mean	Grouping	
2‐year follow‐up	170	75,3109	A	
1‐year follow‐up	176	74,7021	A	
5‐year follow‐up	90	72,8136	A	
6‐month follow‐up	182	66,5738		B

*Note*: Means that do not share a letter are significantly different.

**Table 13:** Oxford knee score Tukey pairwise comparisons: Visit grouping information using the Tukey method and 95% confidence.

Visit	*N*	Mean	Grouping		
2‐year follow‐up	170	43,6765	A		
5‐year follow‐up	90	43,6556	A		
1‐year follow‐up	177	43,2733	A		
6‐month follow‐up	185	41,7401		B	
Pre‐Op	206	22,9320			C

*Note*: Means that do not share a letter are significantly different.

**Table 14:** OKS.

	Pre‐op	6 months	1 year	2 years	5 years
Count	206	185	176	170	90
Average	22.9	41.7	43.3	43.7	43.7
Median	23.0	43.0	45.0	46.0	45.0
SD	6.5	5.8	5.9	5.0	5.0
Min	4	19	18	22	25
Max	37	48	48	48	48

Abbreviations: OKS, Oxford knee score; SD, standard deviation.

**Table 15:** KOOS.

Pain						Symptoms					
	Pre‐op	6 months	1 year	2 years	5 years		Pre‐op	6 months	1 year	2 years	5 years
Count	206	184	179	170	90	Count	206	184	179	170	90
Average	47.5	88.6	90.8	91.3	92.3	Average	53.0	83.9	87.6	89.9	89.1
Median	47.2	94.4	97.2	97.2	97.2	Median	53.6	89.3	92.9	92.9	92.9
SD	14.5	13.3	13.3	13.8	10.2	SD	16.0	13.8	13.0	12.5	10.9
Min	3	31	36	39	53	Min	0	39	21	43	50
Max	86	100	100	100	100	Max	93	100	100	100	100

ADL						Sport					
	Pre‐op	6 months	1 years	2 years	5 years		Pre‐op	6 months	1 year	2 years	5 years
Count	206	184	179	170	90	Count	206	184	179	170	90
Average	50.7	89.1	91.3	92.9	91.6	Average	19.2	61.3	71.3	72.5	75.6
Median	50.0	94.1	95.6	97.1	97.1	Median	15.0	67.5	75.0	80.0	82.5
SD	15.4	12.5	12.5	12.2	11.0	SD	18.4	29.4	26.8	25.5	24.8
Min	0	31	26	49	53	Min	0	0	0	5	5
Max	91	100	100	100	100	Max	90	100	100	100	100

QOL											
	Pre‐op	6 months	1 years	2 years	5 years						
Count	206	184	179	170	90						
Average	22.8	75.4	81.6	81.7	82.8						
Median	18.8	81.3	87.5	87.5	87.5						
SD	17.2	20.2	19.6	21.2	20.0						
Min	0	13	13	19	19						
Max	81	100	100	100	100						

Abbreviations: KOOS, knee injury and osteoarthritis outcome score; QOL, quality of life; SD, standard deviation.

MCID and PASS responder rates were high across all PROMs and timepoints, exceeding 77% for PASS and 90% for MCID from early follow‐up onward, with stable or improving trends over time. Adjustment for potential confounders did not substantially modify the findings. None of the covariates demonstrated a statistically significant association with MCID or PASS thresholds, except for age, which was associated with PASS achievement for the KOOS at 2 years (OR = 1.07, *p* = 0.024). Even under worst‐case assumptions, PASS and MCID responder rates at 5 years remained moderate‐to‐high, and the overall conclusions were unchanged (Tables 16 and 17).

**Table 16:** PASS analysis.

PASS 70 [38]	KOOS pain 6 months	KOOS pain 1 year	KOOS pain 2 years	KOOS pain 5 years
Reached PASS	80%	84%	85%	88%
Below PASS	20%	16%	15%	12%
PASS 33 [14]	OKS pain 6 months	OKS pain 1 year	OKS pain 2 years	OKS pain 5 years
Reached PASS	91%	93%	94%	93%
Below PASS	9%	7%	6%	7%
PASS 40.6 [28]	FJS 6 months	FJS 1 year	FJS 2 years	FJS 5 years
Reached PASS	87%	88%	83%	77%
Below PASS	13%	12%	17%	23%

**Table 17:** MCID analysis.

MCID 7 [14]	OKS pain 6 months	OKS pain 1 year	OKS pain 2 years	OKS pain 5 years
Reached MCID	91%	94%	96%	98%
Below MCID	9%	6%	4%	2%
MCID 8 [38]	KOOS pain 6 moths	KOOS pain 1 year	KOOS pain 2 years	KOOS pain 5 years
Reached MCID	98%	97%	98%	100%
Below MCID	2%	3%	2%	0%

### Adverse events

There have been 51 adverse events reported since the beginning of the study. A full list of adverse events is listed in Table 18. One (2.0%) adverse event occurred intraoperatively and 50 (98.0%) occurred postoperatively. No events were categorised as unanticipated device‐related events.

**Table 18:** Adverse events (*n* = 51).

Count	Relationship to device	Relationship to surgery	Event	Severity
*N* = 11	Not related	Not related	Myocardial infarction, migraine, distal hamstring/pes insertion tendinitis, MCL strain, increased surgical knee pain (*n* = 3), superficial stitches on surgical knee, fall/pain after fall on surgical knee (*n* = 2), knee pain and joint narrowing on lateral compartment of surgical knee	Mild
*N* = 1	Not related	Probably	Post‐op pain	
*N* = 1	Not related	Definitely	Hematoma	
*N* = 1	Unlikely	Unlikely	Knee pain/quadricep weakness	
*N* = 1	Unlikely	Definitely	Fall and partial opening at incision	
*N* = 16	Not related	Not related	Torn shoulder rotator cuff, patellar tendon and pes anserine, distal radius fracture, long term hospitalisation due to whipple procedure, aortic valve replacement, myocardial infarction, fall/pain after fall on surgical knee (*n* = 2), pain in surgical knee (*n* = 2), blood clot, DVT, swelling in surgical leg, *pain with increased activity (*n* = 2), *increase in pain and onset of OA in lateral compartment	Moderate
*N* = 2	Not related	Unlikely	Surgical side quadricep weakness, rash	
*N* = 1	Not related	Probably	Postanaesthesia headache	
*N* = 3	Not related	Definitely	Increased pain/swelling in surgical knee, partial MCL laceration, stitch abscess	
*N* = 2	Unlikely	Unlikely	Pain in medial surgical knee (*n* = 2)	
*N* = 8	Not related	Not related	DVT, cranial nerve damage, myocardial infarction, rupture aortic aneurysm, various cancers (*n* = 2), stroke, achiness in surgical knee	Severe
*N* = 2	Not related	Definitely	Medication reaction, *Infection	
*N* = 2	Unlikely	Not related	Surgical knee pain (*n* = 2)	

*Note*: *AE resulted in revision.

Abbreviations: DVT, deep vein thrombosis; MCL, medial collateral ligament.

There were 6 events (2 events mild, 3 events moderate and 1 event severe) reported as adverse events definitely related to the surgery that did not result in revision. The two mild adverse events were a fall and a hematoma; both resolved with conservative management. The three moderate adverse events were surgical knee pain/swelling, a partial medial collateral ligament (MCL) laceration and a stitch abscess. The knee pain/swelling occurred 4 days postoperatively. The patient was screened for a deep vein thrombosis (DVT), which was negative, with symptoms resolving less than a month later. The partial anterior third MCL laceration was due to a retractor dislodgement during the tibia resection that was treated with primary repair and postoperative hinged brace and partial weight bearing for 6 weeks. Stability was assessed clinically and with valgus stress X‐ray, which showed full healing of the repair. The stitch abscess occurred 1 month postoperatively and resolved after 10 days of Keflex. The one severe adverse event was a medication reaction that occurred 2 days postoperatively and resolved with a visit to the ER and treatment with Benadryl. All six of these events have resolved without further symptoms.

Twelve participants reported postoperative pain in their operative knees that did not require revision. Sixty‐eight participants' knee pain has resolved, and five participant's knee pain is ongoing. There were four adverse events reported that correlated to the four revisions that occurred. Two of the four adverse events that resulted in revision noted pain in the surgical knee with increased activity. One adverse event reported increased pain and onset of osteoarthritis in the lateral compartment. Another adverse event was a deep infection.

### Revisions

Four serious adverse events occurred that resulted in revisions. No association was noted with BMI and revision surgery [51] (Table 19). Three of the four revisions were 70–79 years old (Table 20). One case (25.0%) occurred due to an early infection, 2 (50.0%) revisions occurred due to aseptic loosening (same site) and 1 (25.0%) occurred due to advancing osteoarthritis in the lateral compartment. The infection case was diagnosed at first follow‐up, less than 1 month postoperatively and treated with single‐stage irrigation, debridement with polyethylene exchange and antibiotics. The patient went on to full healing without any recurrence. The two aseptic loosening cases and the advancing osteoarthritis case were converted to total knee arthroplasties. The aseptic loosening revisions occurred at 16 and 24 months postoperatively. The investigators at the sites reported loosening of the tibial tray. The advancing osteoarthritis case was revised at 9 months postoperatively, with lateral compartment deterioration. Revision total knee arthroplasty (TKA) for all cases were performed without complication. All cases were revised with short tibial 30 mm stems without constraint. One case required a 5 mm tibial augment.

**Table 19:** Implantation by BMI—Revision rate by BMI group.

BMI	Count	%	Revision rate	%
20.9–24	13	6.3	1	7.1
25–29	57	27.5	1	1.8
30–34	69	33.3	1	1.4
35–39	50	24.2	1	2.0
40–44	14	6.8	0	0.0
45–49.9	4	1.9	0	0.0
Total	207			

Abbreviation: BMI, body mass index.

**Table 20:** Implantation by age—Revision rate by age group.

Age	Count	%	Revision rate	%
43–49	10	4.8	1	10.0
50–59	41	19.8	0	0.0
60–69	96	46.4	0	0.0
70–79	51	24.6	3	5.8
80–85	9	4.3	0	0.0
Total	207			

### Survivorship

The study demonstrated excellent survivorship at 2 and 5 years. With a total of four revisions the 2‐year survivorship was 98.5%, with 204 implants surviving. Although not all the patients have achieved a 5‐year follow‐up at the time of this manuscript, there were no further revisions to date between the 2‐ and 5‐year follow‐up. For this cohort of patients, Kaplan Meier Survivorship for the respective time points calculated a survival probability of 99% at 1‐year, 98.5% at 2‐year and 98.1% at 5‐year (Table 21).

**Table 21:** Kaplan–Meier survivorship.

95.0% normal CI						
Time	Number at risk	Number failed	Survival probability (%)	Standard error	CI lower (%)	CI upper (%)
6‐ month	205	1	99.5	0.0048192	98.6	100.0
1‐year	203	1	99.0	0.0068154	97.7	100.0
2‐year	201	1	98.5	0.0083472	96.9	100.0
5‐year	103	1	98.1	0.0096385	96.2	99.9

Abbreviation: CI, confidence intervals.

## DISCUSSION

UKA has been shown to provide better PROs compared to TKA [2, 30], and studies have demonstrated that patients undergoing UKA by high‐volume surgeons exhibit comparable medium‐term survival rate to those of patients undergoing TKA [44]. At the time of surgery, authors have suggested potentially 50% of knee replacement patients have only one compartment affected by the pathology [47] and the anterior cruciate ligament is reported to be functional in more than 50% of the cases [12]. UKA may be a valuable alternative to total knee replacement for many patients, with the benefit of preserving knee proprioception and healthy anatomic structures [25], while achieving better clinical outcomes (knee mobility, ROM and activity level [19, 42, 45]). Much of these benefits can be attributed to a less invasive procedure with inherently lower perioperative risks and shorter hospital stay with a faster recovery [3, 10].

Our study is in line with recent reported outcome studies [4, 31]. This study demonstrated significantly improved patient‐reported outcomes that were well sustained for 5 years. The KOOS Pain and ADL subcategory demonstrated the earliest gain of improvement at 6 months and sustained at 5 years. The OKS, FJS, KOOS Sport, QOL had significant improvement from preop to 6 months with further improvement at 1 year and were also well maintained through 5 years. Even at the 5‐year midterm follow‐up, a trend toward a further small improvement was seen in the average scores for the QOL, sport and pain scores. This study continues to support the more recent literature that partial knee replacement can return patients to a high level of function and sport as well as improved QOL.

Total knee arthroplasty can also successfully treat isolated medial knee osteoarthritis. While total knee replacement generally also shows improvement in most metrics at midterm follow‐up [25, 40], most of these studies also report much higher complication rates, such as infection, instability and arthrofibrosis [1, 5]. Infection remains one of the most debilitating and morbidity‐producing complications. Authors have seen that if this complication should occur after total knee replacement, many of these patients require multiple more invasive surgeries, implant extraction and long‐term antibiotic suppression. Outcome scores and overall function are generally poor [8, 35]. In a review of over 11,000 cases, Koh et al. [24] found infection to be the main cause of failure for current knee arthroplasty. Of the repeat surgeries in this study, only one infection was reported in 207 cases, which was treated with an irrigation, debridement and polyethylene exchange with primary component retention. As the number of arthroplasty cases increases over the next decade, the costs of treatment of postoperative infection will increase proportionately, with estimated costs annually in the United States to be $1.85 billion by 2030 [41]. Septic TKA two‐stage revision can be four times the cost of standard knee arthroplasty [35]. Adding indirect costs to the total increases the episode of care dramatically [36]. Iqbal and authors noted the burden of costs extends globally, with similar findings of 4.5x increased costs in developing countries [18]. Revision TKA for infection is also reported to have lower success rates compared to revision for other causes [7]. It becomes prudent for the surgeon to weigh these considerations when recommending total versus partial knee arthroplasty, especially for patients with unicompartmental disease. Studies confirm a significantly lower complication rate including infection for UKA. This study was significant for a very low infection rate of 0.5% and supports the utilisation of unicompartmental arthroplasty, particularly patients at higher risk for infection.

Although this study recorded any and all adverse events, very few were related to the surgery. Three mild AEs were seen, three moderate and one severe. Six of these were resolved uneventfully, and one case required a visit to the emergency room and was treated with medication and discharged the same day. No manipulations were necessary, nor any revision for arthrofibrosis. No thromboembolic complications related to the surgery were reported. Instability is rarely reported in the partial knee replacement literature and was not a cause for revision in this study, unlike total knee replacement where this still remains a common cause for revision [27, 37].

One of the most impactful features of partial knee replacement is achieving a successful surgery as measured by pain relief and patient functional improvement with a very low complication rate. This gives the surgeon a viable and safer option for those patients that fall into a higher perioperative risk profile with unicompartmental disease. There may even be an argument to consider greater patellofemoral osteoarthritis in light of the lower perioperative risks if the higher‐risk patient is made aware of the risks and benefits. This can also be supported by results from Plancher, who reported equal outcomes and survivorship in medial UKA for patients with grade 3–4 lateral facet patellofemoral osteoarthritis versus normal.

Much of the criticism of partial knee replacement has traditionally been the higher revision and complication rates as seen in the older literature [31]. Ji et al. states a complication rate of 9.8% (24/246) [22]. With only 3 revisions that resulted in TKA conversions, this study demonstrates promise for a very high survivorship at midterm follow‐up, with a 98.1% survivorship at 5 years. The author's philosophy to unicompartmental replacement requires a balanced aligned resection approach. The balancing during the surgery requires avoidance of overcorrection [50]. An understanding of the preoperative mechanical axis position of the knee in addition to the amount of potential correction seen by the stress x‐rays, which is bringing the medial collateral ligament to full length. Using the combination of the long leg x‐rays to determine the preoperative mechanical axis starting point and adding the amount of medial compartment opening seen on stress x‐ray gives the surgeon critical information to recognise preoperatively the cases that have greater potential for overcorrection and ability to mitigate this intraoperatively. To our knowledge this is the first description of different types of stress x‐rays depending on the amount of opening of the medial relative to the lateral compartment. This is intended to give the surgeons an awareness preoperatively of cases that may have potential to overcorrect. The value of this can be seen comparatively, a case that is in less than 5 degrees of varus with a type III stress x‐ray has a much greater potential for overcorrection into valgus, than a case starting in 10 degrees of preoperative varus and a Type I stress x‐ray. In this study, one case underwent early revision for lateral compartment degeneration. The case was a Type III stress x‐ray, which was corrected from 171 degrees varus to 178 degrees varus. The increased 7‐degree correction likely contributed to the lateral compartment failure at 9 months. Type III cases with five degrees varus or less may be treated with UKA but should be addressed by very experienced surgeons. The authors strongly emphasise the importance of obtaining the full x‐rays series, valgus stress and long leg preoperatively in all cases and feel this should be a requirement for proper preoperative planning. A greater understanding of alignment may decrease one of the main causes for UKA revision, opposite compartment failure.

Nonprogressive radiolucent lines were noted on 5‐year follow‐up x‐rays. These remained stable through 5 years, with the exception of the two revisions for tibial component aseptic loosening, where the radiolucency progressed to adjacent zones. Although, for the rest of the patients at the 5‐year follow‐up no change of component position was noted. No association was seen with BMI and revision. Three cases in this study were revised within 2 years of initial surgery were, as the literature has suggested, likely due to poor patient selection or surgical technique. Jeer described, even for experienced surgeons, technical errors may occur such as overcorrection, poor pin placement and poor cementation as causes for complications including opposite compartment failure and aseptic loosening [20]. In this patient series overcorrection and aseptic loosening were the main reasons for revision. All of the revisions to total knee were performed without constraint, with one case requiring a small tibial augment.

The older revision rates reported for UKA need to be considered in light of the time the data was published. Instrumentation was unforgiving and implants were less anatomic. Chattellard reported higher failure rates with slope changes greater than 2 degrees, as well as greater than 2 mm joint height change [6]. The inability to accommodate necessary changes for proper balancing were largely due to older instrumentation with fixed resections, making any adjustments with older instrumentation limited to none. Authors have suggested that increased tibial implant slope of 7 degrees carries potential for attritional ACL rupture [15]. To address patients with higher preoperative slope while avoiding increased tibial implant slope, requires the ability to resect more posterior femur to avoid an excessively tight flexion gap. Instrumentation systems such as the one used in this study must accommodate variable resections in 1‐mm increments of the distal and posterior femur as well as the tibia. The system used for this study allows independent flexion and extension balancing, avoiding instability and more importantly overcorrection. With these features, issues such as slope change and joint line change are possible to allow proper balancing and did not affect the patient‐reported outcomes or survivorship as seen in this study.

In addition to a lack of control group and absence of multivariable adjustments, another weakness to this study was the experience of the investigators in partial knee replacement. While the investigators have 15–30 years of experience in orthopaedic operations, the investigator's unicompartmental practice ranged from 30% to 2% as a percentage of surgeon total arthroplasty volume [26, 33]. Complication rates were higher for the surgeons with lower practice UKA volume 4% versus 0.75% for the surgeons with higher uka practice volume. The purpose of the variation was to explore whether the procedure could be adopted by surgeons less familiar with the procedure and to make the results more representative of common orthopaedic surgery practice. Another weakness is the different x‐ray technicians at the sites performing the stress x‐rays. The variety of technicians may produce a different percentage of the different stress types. Devices are available to standardise the x‐ray but these were not incorporated into this study. Performing the stress x‐ray is typically similar to a valgus stress clinical exam to assess the function of a patient's MCL, with the goal to simply bring the ligament under full tension and has been described by several authors [11, 49]. Another weakness for this study included the small number of patients. The numbers were enough to achieve statistical significance for patient‐reported outcome results. Alignment of nine patients were transitioned from varus/neutral to valgus and 11 patients from varus to neutral. Not all of these patients have achieved a 5‐year follow‐up. This may have an effect on the results and survivorship at 5 years. We plan to continue to report the results as all the patients achieve 10‐year survivorship.

In conclusion, this study supports the use of UKA for properly indicated patients. Since this study is still active, 5 year survivorship is reported on the available data which shows excellent promise at 98.1%. Overall complication rates related to the surgery and implant as well as revision rate in this study was exceptionally low at 5 years to date. To improve outcomes and avoid opposite compartment failure, it is essential to include long‐leg and valgus stress x‐rays as an essential part of the preoperative workup. Implant systems, such as the one used in this study, that allow surgical flexibility and independent balancing are critical to optimise long‐term surgical success. All patient‐reported outcomes showed statistically significant improvement that continued through 5 years. Nonprogressive radiolucent lines were occasionally seen on postoperative x‐rays but were not associated with a decrease in patient‐reported outcomes or survivorship. Further follow‐up is necessary to determine the long‐term durability of the implant and surgical technique.

## AUTHOR CONTRIBUTIONS

**Akbar Nawab**: Writing—original draft; investigation; data curation; methodology. **Patricia Bartelt**: Writing—original draft; data curation; formal analysis; project administration; visualisation. **Ryan Molli**: Writing—review and editing; investigation; methodology. **J Mandume Kerina**: Writing—review and editing; investigation; methodology. **Samuel Wellman**: Writing—review and editing; investigation. **Dean Sukin**: Writing—review and editing; investigation. **Stefano Biguzzi**: Writing—original draft; data curation; formal analysis; visualisation. **Colleen Nawab**: Project administration. **Daniel Hass**: Project administration. **Ashton Dukes**: Project administration. **Anthony Robins**: Writing—review and editing; data curation; methodology.

## CONFLICT OF INTEREST STATEMENT

All support for the present manuscript, including funding, provision of study materials, medical writing and article processing charges, was provided by Medacta. Author Dr. A. Nawab receives royalties or licences from Medacta and has received consulting fees from the company. Additionally, the author has received payment or honoraria from Medacta for lectures, presentations, speakers bureaus, manuscript writing and educational events. The author serves on an advisory board for Metro Specialty Surgery Center. Furthermore, the author holds leadership or fiduciary roles with Ellis and Badenhausen Orthopaedics and Metro Specialty Surgery Center. Author Bartelt is a paid employee of Medacta USA. Author Dr. Molli receives royalties and consulting fees from Medacta. Author Dr. Kerina receives royalties or licences, consulting fees, and payment or honoraria for lectures, presentations, speakers bureaus, manuscript writing or educational events, all from Medacta. Author Dr. Wellman has received grants or contracts from Stryker, DuPuy Synthes, Smith & Nephew, Zimmer Biomet, and Medacta. The author receives royalties and consulting fees from Total Joint Orthopaedics and has received payment or honoraria from Zimmer Biomet and Total Joint Orthopaedics for lectures, presentations, speakers bureaus, manuscript writing or educational events. The author holds a leadership or fiduciary role with the Hip Society and owns stock or stock options in Joint Development LLC. Author Dr. Sukin has no financial or nonfinancial interests to disclose. Author Biguzzi is a paid employee of Medacta International SA. Author Dr. C. Nawab has no financial or nonfinancial interests to disclose. Author Haas has received payment or honoraria from Super Spine for lectures, presentations, speakers bureaus, manuscript writing or educational events. Author Dukes is a paid employee of Ellis & Badenhausen Orthopedics. Author Dr. Robins receives royalties from Medacta and consulting fees from both Medacta and Moximed. The author has received payment or honoraria from Medacta for medical education activities. Additionally, the author serves as a medical monitor on the data safety monitoring board for Moximed.

## ETHICS STATEMENT

WCG IRB (Nawab, Molli, Sukin): 20171512. Informed consent was obtained from all individual participants included in the study.

## Data Availability

The data that support the findings of this study are available on request from the corresponding author. The data are not publicly available due to privacy or ethical restrictions.
